# Pregnancy-related morbidity and risk factors for fatal foetal outcomes in the Taabo health and demographic surveillance system, Côte d’Ivoire

**DOI:** 10.1186/s12884-018-1858-2

**Published:** 2018-06-07

**Authors:** Siaka Koné, Eveline Hürlimann, Nahoua Baikoro, Daouda Dao, Bassirou Bonfoh, Eliézer K. N’Goran, Jürg Utzinger, Fabienne N. Jaeger

**Affiliations:** 1 Centre Suisse de Recherches Scientifiques en Côte d’Ivoire, 01 BP 1303, Abidjan, 01 Côte d’Ivoire; 2 INDEPTH Network, Accra, Ghana; 3 Swiss Tropical and Public Health Institute, Basel, Switzerland; 4 University of Basel, Basel, Switzerland; 5 Unité de Formation et de Recherche Biosciences, Université Félix Houphouët-Boigny, Abidjan, Côte d’Ivoire

**Keywords:** Côte d’Ivoire, Early neonatal death, Foetal health, Health and demographic surveillance system, Miscarriage, Mother and child health, Perinatal mortality, Pregnancy, Stillbirth

## Abstract

**Background:**

Reliable, population-based data on pregnancy-related morbidity and mortality, and risk factors for fatal foetal outcomes are scarce for low- and middle-income countries. Yet, such data are essential for understanding and improving maternal and neonatal health and wellbeing.

**Methods:**

Within the 4-monthly surveillance rounds of the Taabo health and demographic surveillance system (HDSS) in south-central Côte d’Ivoire, all women of reproductive age identified to be pregnant between 2011 and 2014 were followed-up. A questionnaire pertaining to antenatal care, pregnancy-related morbidities, delivery circumstances, and birth outcome was administered to eligible women. Along with sociodemographic information retrieved from the Taabo HDSS repository, these data were subjected to penalized maximum likelihood logistic regression analysis, to determine risk factors for fatal foetal outcomes.

**Results:**

A total of 2976 pregnancies were monitored of which 118 (4.0%) resulted in a fatal outcome. Risk factors identified by multivariable logistic regression analysis included sociodemographic factors of the expectant mother, such as residency in a rural area (adjusted odds ratio (aOR) = 2.87; 95% confidence interval (CI) 1.31–6.29) and poorest wealth tertile (aOR = 1.79; 95% CI 1.02–3.14), a history of miscarriage (aOR = 23.19; 95% CI 14.71–36.55), non-receipt of preventive treatment such as iron/folic acid supplementation (aOR = 3.15; 95% CI 1.71–5.80), only two doses of tetanus vaccination (aOR = 2.59; 95% CI 1.56–4.30), malaria during pregnancy (aOR = 1.94; 95% CI 1.21–3.11), preterm birth (aOR = 4.45; 95% CI 2.82–7.01), and delivery by caesarean section (aOR = 13.03; 95% CI 4.24–40.08) or by instrumental delivery (aOR = 5.05; 95% CI 1.50–16.96). Women who paid for delivery were at a significantly lower odds of a fatal foetal outcome (aOR = 0.39; 95% CI 0.25–0.74).

**Conclusions:**

We identified risk factors for fatal foetal outcomes in a mainly rural HDSS site of Côte d’Ivoire. Our findings call for public health action to improve access to, and use of, quality services of ante- and perinatal care.

## Background

While major progress has been made over the past 15 years to improve population health and wellbeing [1], maternal and neonatal mortality still remain high, particularly in the poorest countries, where stillbirths occur frequently [2, 3]. Pregnancy-related morbidity and complications during childbirth have a significant negative impact on the foetus and may result in fatal outcomes [4, 5]. Fatal foetal outcome include miscarriage (early foetal death), stillbirth, and early neonatal mortality. Miscarriage is usually considered as pregnancy loss of a foetus that did not yet reach the gestational age of 23 weeks or a weight inferior to 500 g in case gestational age is unknown [6, 7]. For international comparison, however, the World Health Organization (WHO), recommends to use the definition of a child born dead of at least 28 weeks of gestational age with a birth weight over 1000 g or a birth size ≥35 cm to discriminate between miscarriage and stillbirth [7]. Neonatal death, considered death of a live birth within the first 28 days of life, is further subdivided into early (up to 7 days of life) and late neonatal death (8–28 days of life) [7]. For 2015, there were an estimated 2.12 million stillbirths, and an additional 2.03 million early neonatal deaths, thus death occurring within 7 days after a live birth [2]. Stillbirth and neonatal death also come at a substantial direct, indirect, and tangible cost, not only to the mothers and fathers and their family, but also staff who care for them and the society at large [8].

There is a paucity of data in low-income countries pertaining to the incidence of foetal deaths, partially explained by the lack of designated health policies, programmes, and adequate surveillance platforms [9]. While a panoply of maternal and foetal factors are recognised for stillbirths, estimates on their importance in contributing to a fatal foetal outcome are based on theoretical constructs using mathematical modelling of prevalence estimates of “known” associated risk factors in the population [10]. However, these estimates usually lack accuracy, as data are poorly reported or not reported at all. The majority of foetal and early neonatal deaths are estimated to occur in South Asia and sub-Saharan Africa [11, 12]. Yet, in these regions, there is underreporting due to a lack of data repositories on neonatal mortality, stillbirth, miscarriage, and induced abortion. In general, foetal deaths are not routinely reported and included in essential population statistics of many low- and middle-income countries [13]. In Côte d’Ivoire, for example, more than 40% of deliveries occur outside a health facility, and hence, risk factors for fatal foetal outcomes remain to be thoroughly investigated [14]. Moreover, the often found preference for traditional birth assistance and non-facility-based deliveries, paired with reluctance towards the use of antenatal care, may put expectant mothers and their unborn babies at additional risks [15–17]. There is a pressing need for quality information regarding pregnancy-related morbidity and health system use in order to better understand risk factors of pregnancy complications, so that foetal and maternal death rates can be lowered [18].

The Taabo health and demographic surveillance system (HDSS) in south-central Côte d’Ivoire – like other members of the International Network for the continuous Demographic Evaluation of Populations and their Health (INDEPTH) – documents pregnancy-related morbidity and other vital statistics in a distinct geographic region at the household level [19]. We investigated the most important pregnancy-related morbidities and factors associated with fatal foetal outcomes in the Taabo HDSS, including all pregnancies starting and ending between January 1, 2011 and December 31, 2014. Our findings, although not representative for the whole of Côte d’Ivoire, provide an evidence-base and allow for priority setting in order to improve maternal and neonatal health in rural areas of Côte d’Ivoire and elsewhere in sub-Saharan Africa.

## Methods

### Study area and design

This study was conducted within the Taabo HDSS [20, 21]. The Taabo HDSS includes one small town (Taabo Cité), which is the centre of the department of Taabo that also holds the only small hospital for the surveillance zone. There are 13 main villages with more than 100 associated hamlets. The latter are settlements usually consisting of a group of households constructed close to agricultural exploitation sites, rather isolated, and not yet officially considered a village by the territorial administrative authority mainly due to its small population size (< 500 inhabitants). Meanwhile, there is a primary health care centre in all of the 13 villages, 10 of which were fully operational with an assigned nurse and three are managed by trained community-health workers (CHWs) not yet being entirely functional. In the hamlets, no basic primary care is available but CHWs that are part of the hamlet’s population may be approached for advice before seeking formal care. Basic antenatal care is provided at the general hospital of Taabo and all nurse-led operational health centres; thereof four villages also host professional midwives who offer their service. With regard to emergency obstetric care (EOC), such as caesarean section and instrumental delivery (e.g. *forceps* delivery), the first is supposed to be only done at the general hospital of Taabo where an operating block is available, while instrumental deliveries may also being performed as emergency measure by midwives. For women delivering in a health facility, referral to a better equipped medical centre in case of complications is decided and an official transfer statement provided by the respective midwives. However, the actual transport remains to be organised by the women’s relatives due to a lack of ambulances (only available in Taabo-Cité and the village of Kotiéssou). The costs for antenatal care and birth assistance are not standardised and thus difficult to be estimated. Certainly the costs increase with the level of proficiency of the service providers and depend on whether facilities are public or private [17]. Since 2011 antenatal care and delivery are by national policy free of charge, however additional costs for health seeking by expectant mothers are common [22]. Of note, in primarily rural areas such as the Taabo HDSS many women, especially when it is their first child, still tend to spend the last trimester of their pregnancy close to their relatives that may live in more remote areas. The place of labour and child birth may thus differ in many cases from the actual residence of the expectant mothers.

The objective of this study was to assess pregnancy-related morbidities and risk factors for a fatal foetal outcome. All women of reproductive age (15–49 years) whose pregnancy started and ended between January 1, 2011 and December 31, 2014 were included. Each household of the Taabo HDSS is visited at least three times a year for detailed surveillance of vital events (i.e. birth, death, in-migration, out-migration, and pregnancy). New pregnancies were systematically listed and followed-up longitudinally. The status and potential negative events related to pregnancy were registered by trained field-enumerators. Miscarriage, stillbirth, and live birth were registered as pregnancy outcomes.

Each woman identified with a new pregnancy was interviewed with a pre-tested questionnaire with an emphasis on pregnancy status, estimated date of last menstrual period (LMP), and number of earlier pregnancies and births. Furthermore, in relation to potential negative consequences of a pregnancy, a standardised INDEPTH questionnaire on pregnancy-related morbidity was administered by field-enumerators to expectant mothers [23]. Sociodemographic information from women becoming pregnant during the 4-year observational study was readily available from the Taabo HDSS database [20].

### Statistical analysis

**Fig. 1:**
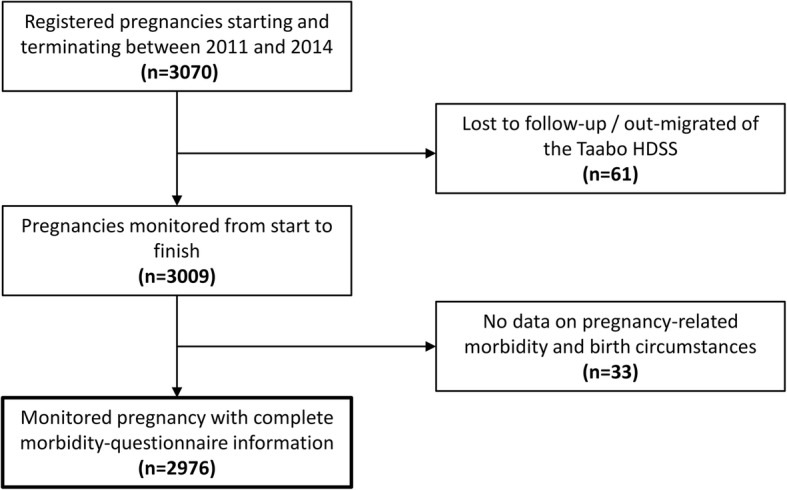
Flow chart indicating all pregnancies registered and monitored between 2011 and 2014 in the Taabo HDSS and final study sample comprising complete data for analysis

Data were double-entered, cross-checked, and managed using a household registration system implemented in Windev version 12.0 (PC Soft; Montpellier, France) [24]. All statistical analyses were performed in Stata version 12.0 (StataCorp; College Station, TX, USA). Data records from pregnant women with complete sociodemographic, pregnancy-related morbidity, and birth circumstances information that were not lost to follow-up were considered for analysis (Fig. 1).

The primary outcome variable was defined as fatal foetal outcome and applied to all pregnancies that resulted in stillbirth, miscarriage, or early neonatal death using WHO definitions (i.e. dead born with gestational age higher or lower than 28 weeks, or death within the first 7 days after birth) [7]. Assessed explanatory variables for relationship analysis included (i) sociodemographic characteristics of the expectant mother; (ii) antenatal care sought; (iii) pregnancy-related morbidities and concomitant health conditions; and (iv) circumstances of delivery reported. Socioeconomic status was determined using a household-based asset approach and principal component analysis (PCA) with stratification into wealth tertiles (i.e. poorest, poor, and least poor) [25].

Gestational age at birth was calculated based on the first day of LMP and date of birth. Preterm birth was defined as gestational age <  37 weeks [26] or as perceived “shorter than normal” (i.e. estimated duration of < 8 months) preterm birth in women not able to provide reliable information on LMP (about 8% of all expectant mothers).

χ^2^ test statistics were used to investigate significant univariate differences between mothers whose pregnancy resulted in live birth compared to a fatal outcome for the aforementioned explanatory variables. Univariate and multivariable logistic regression analyses were performed to identify significant relationships between fatal foetal outcome and covariates. In order to address estimation bias from fatal foetal outcome being a rare event, penalized maximum likelihood logistic regression models, as proposed by Firth, were used [27, 28]. Results were presented as odds ratios (ORs) and 95% confidence interval (CI). Differences and relationships with a *p*-value below 0.05 were considered as statistically significant. The multivariable regression model was built using a stepwise elimination approach, excluding explanatory variables at a significance level of 0.20 or higher. Sociodemographic factors known to have negative consequences on birth outcome (e.g. age, socioeconomic status, and residency of the mother) from earlier studies were included in the final model [29].

## Results

### Study sample and sociodemographic characteristics

From a total of 3070 pregnancies registered in the Taabo HDSS over a 4-year period, 3009 were monitored from start to finish. Sixty-one pregnant women out-migrated from the surveillance area or were lost-to-follow-up. Another thirty-three expectant mothers had no information on pregnancy-related morbidity and birth circumstances, as assessed during the questionnaire interview (Fig. 1). Taken together, 2976 monitored pregnancies had complete data records, and hence, were considered as final study sample. Within these fully monitored pregnancies 2858 (96.0%) resulted in live birth. The remaining 118 (4.0%) were fatal foetal outcomes and are the subject of the current in-depth analysis.

There were 63 (53.4%) stillbirths (gestational age ≥ 28 weeks), 35 (29.7%) early neonatal deaths (live birth with death within the first 7 days), and 20 known (16.9%) miscarriages (gestational age estimated < 28 weeks). Our findings translate into a miscarriage rate of 6.7, a stillbirth rate of 21.2 and an early neonatal death rate of 11.8 per 1000 births. The latter two rates are summed up into a perinatal mortality of 32.9 per 1000 births. Only a small proportion of the expectant mothers were urban residents (12.8%), while the large majority lived in rural areas (87.2%). More than four out of five mothers were below the age of 35 years (83.3%). Women with no or low educational attainment represented 62.8% and 28.8%, respectively, of all pregnancies investigated. Most of the women lived with a partner, either legally married (51.0%) or in a common-law relationship (40.5%). With regard to religion, more than half of the mothers reported that they are animist (51.0%), while Muslim and Christians accounted for 40.7% and 7.7%, respectively. Univariate regression analysis revealed no statistically significant association between foetal outcome and sociodemographic characteristics of the expectant mothers, such as age, educational attainment, alphabetisation, socioeconomic and marital status, religion, or residency (Table 1).

**Table 1:** Mother’s sociodemographic characteristics, stratified by foetal outcome and crude ORs for fatal outcome, in the Taabo HDSS in 2011–2014, as assessed by univariate logistic regression analysis

Sociodemographic characteristics	Foetal outcome				
	Live birth^a^	Fatal birth	Total		
	2858 (96.0)	118 (4.0)	2976 (100.0)	OR (95% CI)	*P*-value
Maternal age (years)					
20–34	1994 (69.8)	80 (67.8)	2074 (69.7)	1.00	
≤19	389 (13.6)	16 (13.6)	405 (13.6)	1.05 (0.61, 1.80)	0.861
≥35	475 (16.6)	22 (18.6)	497 (16.7)	1.17 (0.73, 1.89)	0.514
Educational attainment					
Never attended school	1795 (62.8)	75 (63.6)	1870 (62.8)	1.00	
Primary school	823 (28.8)	34 (28.8)	857 (28.8)	0.99 (0.65, 1.50)	0.957
Secondary school or higher	240 (8.4)	9 (7.6)	249 (8.4)	0.90 (0.44, 1.82)	0.763
Literacy					
Literate	578 (20.2)	21 (17.8)	599 (20.1)	1.00	
Illiterate	2280 (79.8)	97 (82.2)	2377 (79.9)	1.17 (0.72, 1.89)	0.520
Socioeconomic status (wealth tertile)					
Least poor	955 (33.4)	35 (29.7)	990 (33.2)	1.00	
Poor	957 (33.5)	36 (30.5)	993 (33.4)	1.03 (0.64, 1.64)	0.915
Most poor	946 (33.1)	47 (39.8)	993 (33.4)	1.35 (0.87, 2.11)	0.184
Marital status					
Unmarried	221 (7.7)	14 (11.9)	235 (7.9)	1.00	
Common-law union	1164 (40.7)	41 (34.7)	1205 (40.5)	0.54 (0.29, 1.01)	0.053
Married	1457 (51.0)	62 (52.5)	1519 (51.0)	0.66 (0.36, 1.18)	0.159
Divorced/widowed	16 (0.6)	1 (0.9)	17 (0.6)	1.39 (0.24, 8.02)	0.714
Religion					
Christian	221 (7.7)	14 (11.9)	235 (7.9)	1.00	
Muslim	1164 (40.7)	41 (34.7)	1205 (40.5)	0.99 (0.66, 1.48)	0.961
Animist	1457 (51.0)	62 (52.5)	1519 (51.0)	0.33 (0.02, 5.46)	0.439
Other religion	16 (0.6)	1 (0.8)	14 (0.6)	0.62 (0.34, 1.15)	0.129
Residency					
Urban	371 (13.0)	11 (9.3)	382 (12.8)	1.00	
Rural	2486 (87.0)	107 (90.7)	2594 (87.2)	1.45 (0.77, 2.73)	0.246

^a^Includes all live births with survival of more than 7 days

*CI* confidence interval, *OR* odds ratio

### Antenatal care, birth circumstances, and foetal outcome

As shown in Table 2, univariate regression analysis revealed several significant associations between fatal foetal outcome and antenatal care and birth circumstances. Antenatal care, such as visits of health care centres during pregnancy, iron and folic acid supplementation, and receipt of two doses of tetanus vaccine were negatively associated with fatal foetal outcome. Women without antenatal care visits (3.7%) were more likely to experience negative consequences on their foetus’ health compared to women going for check-ups (OR = 4.56; 95% CI 2.59–8.03). Women who did not receive two doses of tetanus (55.7%) had a higher odds of a fatal outcome (OR = 3.07; 95% CI 1.96–4.81). Similarly, women who lacked iron and folic supplementation were at higher odds of fatal foetal outcome (OR = 2.37; 95% CI 1.41–3.99).

**Table 2:** Use of antenatal care and birth circumstances in the Taabo HDSS in 2011–2014, stratified by foetal outcome, and crude ORs for fatal outcome from univariate logistic regression analysis

	Foetal outcome				
Characteristics	Live birth^a^	Fatal birth	Total		
	2858 (96.0)	118 (4.0)	2976 (100.0)	OR (95% CI)	*P*-value
Antenatal care					
Antenatal visits					
Yes	2763 (96.7)	102 (86.4)	2865 (96.3)	1.00	
No	95 (3.3)	16 (13.6)	111 (3.7)	4.56 (2.59, 8.03)	< 0.001*
Rubella test					
Yes	138 (4.8)	4 (3.4)	142 (4.8)	1.00	
No	2720 (95.2)	114 (96.6)	2834 (95.2)	1.30 (0.50, 3.37)	0.596
HIV test					
Yes	539 (18.9)	14 (11.9)	553 (18.6)	1.00	
No	2319 (81.1)	104 (88.1)	2423 (81.4)	1.73 (0.98, 3.04)	0.058
Two doses of tetanus vaccination					
Yes	1293 (45.2)	25 (21.2)	1318 (44.3)	1.00	
No	1565 (54.8)	93 (78.8)	1658 (55.7)	3.07 (1.96, 4.81)	< 0.001*
Iron/folic acid supplementation					
Yes	816 (28.6)	17 (14.4)	833 (28.0)	1.00	
No	2042 (71.4)	101 (85.6)	2143 (72.0)	2.37 (1.41, 3.99)	0.001*
Syphilis test					
Yes	425 (14.9)	12 (10.2)	437 (14.7)	1.00	
No	2433 (85.1)	102 (89.8)	2539 (85.3)	1.54 (0.84, 2.83)	0.161
Birth circumstances					
Location during delivery					
HDSS hamlets	299 (10.5)	4 (3.4)	303 (10.2)	1.00	
HDSS village	1682 (58.8)	78 (66.1)	1760 (59.1)	3.11 (1.19, 8.09)	0.020*
Taabo-cité	538 (18.8)	22 (18.6)	560 (18.8)	2.78 (1.00, 7.73)	0.050
Outside Taabo HDSS	339 (11.9)	14 (11.9)	353 (11.9)	2.84 (0.98, 8.28)	0.055
Place of delivery					
Health centre/hospital	1419 (49.6)	63 (53.4)	1482 (49.8)	1.00	
At home	1340 (46.9)	52 (44.1)	1392 (46.8)	0.88 (0.60, 1.27)	0.485
On the way to health centre	22 (0.8)	2 (1.7)	24 (0.8)	2.48 (0.66, 9.40)	0.180
Other	77 (2.7)	1 (0.8)	78 (2.6)	0.43 (0.08, 2.22)	0.315
Birth assistance					
Midwife/doctor/nurse	1390 (48.6)	61 (51.7)	1451 (48.8)	1.00	
Traditional birth assistance	341 (11.9)	13 (11.0)	354 (11.9)	0.69 (0.47, 1.60)	0.651
Alone	61 (2.1)	10 (8.5)	71 (2.4)	3.74 (1.83,7.64)	< 0.001*
Parent/friend	997 (34.9)	33 (28.0)	1030 (34.6)	0.75 (0.49, 1.16)	0.200
Other	69 (2.4)	1 (0.8)	70 (2.3)	0.33 (0.05, 2.42)	0.275
Delivery					
Normal	2801 (98.0)	105 (89.0)	2906 (97.7)	1.00	
Caesarean section	29 (1.0)	8 (6.8)	37 (1.2)	7.65 (3.48, 16.81)	< 0.001*
Instrumental delivery	28 (1.0)	5 (4.2)	33 (1.1)	5.12 (2.02, 13.03)	0.001*
Heavy bleeding during delivery					
No/cannot remember	2301 (80.5)	81 (68.6)	2382 (80.0)	1.00	
Yes	557 (19.5)	37 (31.4)	594 (20.0)	1.90 (1.28, 2.83)	0.002*
Childbirth costs					
Mean amount, in FCFA (min-max)	14,479 (500–996,000)	30,066 (1000–400,000)	15,004 (500–996,000)		
Free of charge	388 (13.6)	31 (26.3)	419 (14.1)	1.00	
Payment of charge	606 (21.2)	22 (18.6)	628 (21.1)	0.45 (0.26, 0.80)	0.006*
Do not know	1864 (65.2)	65 (55.1)	1929 (64.8)	0.44 (0.28, 0.68)	< 0.001*

*CI* confidence interval, *OR* odds ratio

*Includes all live births with survival of more than 7 days

Concerning birth circumstances, it was found that, compared to women who gave birth in hamlets (10.2%), those giving birth in main villages of the Taabo HDSS (59.1%) were at a higher odds of experiencing a fatal outcome (OR = 3.11; 95% CI 1.19–8.09). Home deliveries were not associated with an increased odds for fatal foetal outcome (OR = 0.88, 95% CI 0.60–1.27) compared to facility-based deliveries in univariate analysis. If only vaginal births were considered, excluding EOC such as caesarean section and delivery using instruments, facility- and home-based deliveries showed comparable fatality rates of 35.4 and 37.4 fatal events per 1000 births, respectively. Nevertheless, the place of delivery plays a role if the mother’s residency is taken into account.

**Fig. 2:**
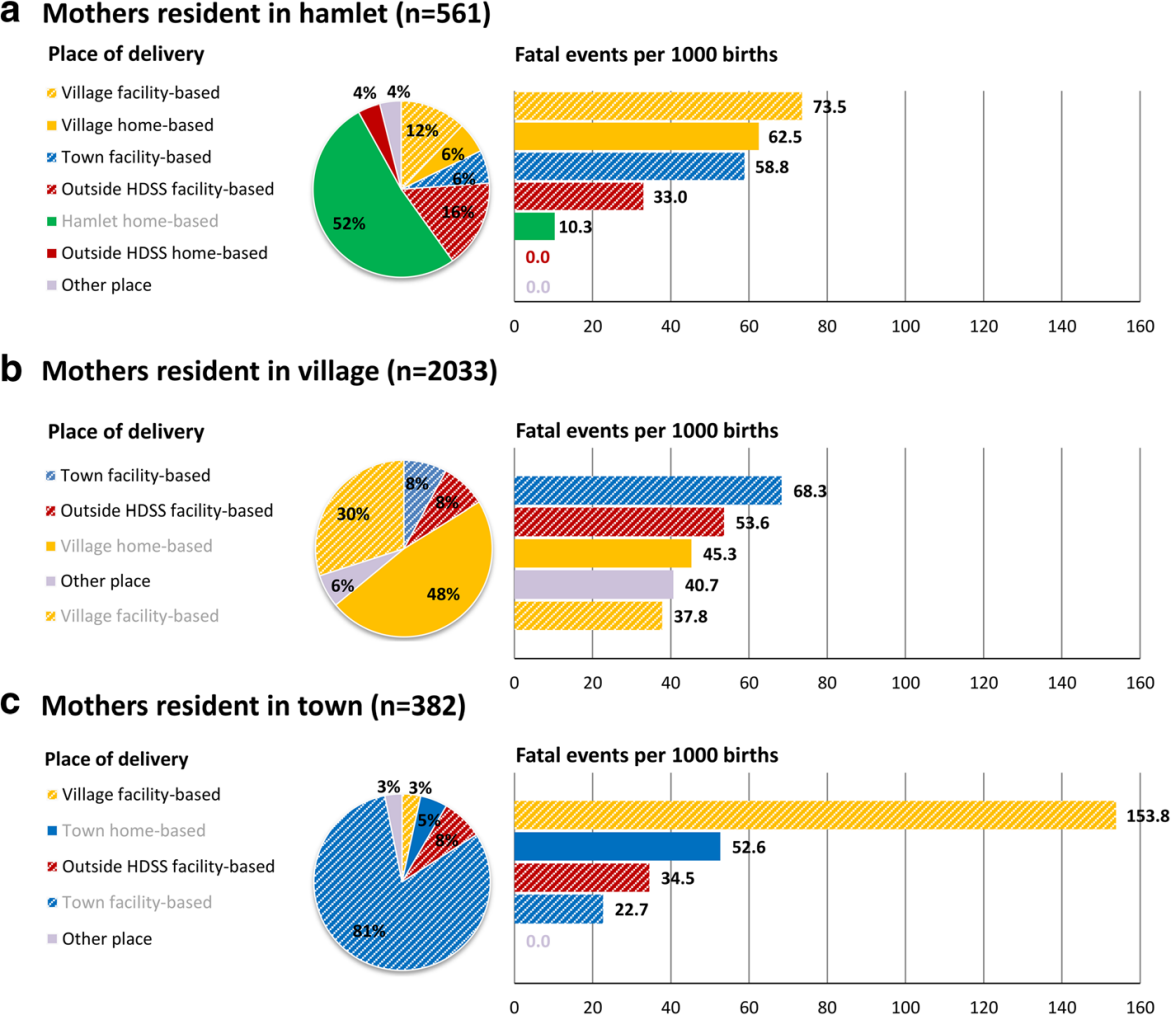
Proportion of place of delivery by residency of the mother and corresponding numbers of fatal events for each place by residency among 2976 pregnancies monitored during a 4-year period in 2011–2014 in the Taabo HDSS. Greyish categories for place of delivery highlight places at the mothers own residency

Figure 2 shows the main places of delivery for women from hamlets, villages, and the urban town of Taabo and provides estimations of fatal outcome rates for each of these places, stratified by the mother’s residence. Women with urban residency had the highest proportion of facility-based deliveries (91.9%), whilst most used the health services of the Taabo general hospital (80.9%). If translated into number of fatal events per 1000 births, the findings further highlight higher fatality rates in facilities for non-resident mothers. For example, mothers from hamlets and the town using health centres of villages had a higher fatality rate compared to mothers from villages using the same health centres (73.5 and 153.8 vs. 37.8 per 1000 births). Similarly, mothers residing in the town had a lower incidence of fatal outcomes, compared to hamlet and village mothers delivering at the town’s hospital (22.7 vs. 58.8 and 68.3 per 1000 births). Women of hamlets (51.9%) and villages (47.8%) showed comparable rates of home deliveries, as defined as non-facility based delivery at own residency. Fatality rates, however, were higher in the village settings (10.3 vs. 45.3 per 1000 births).

**Fig. 3:**
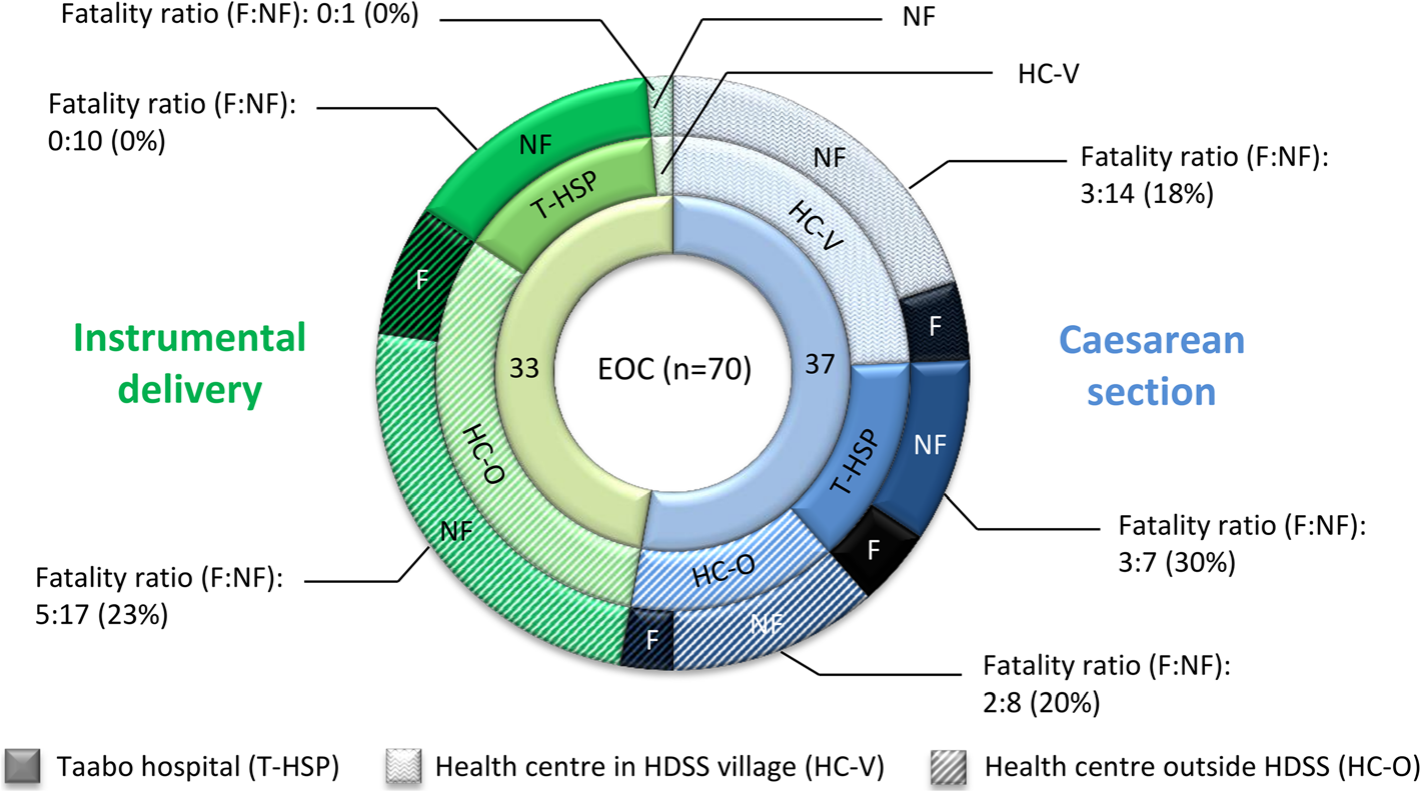
Type of emergency obstetric care (EOC) measures undertaken (*n* = 70) and fatality ratio (number fatal:number non-fatal) and prevalence (%) by type of health care provider as assessed during monitoring of all pregnancies in the Taabo HDSS from 2011 to 2014. 1st ring: type of emergency obstetric care (EOC) (i.e. caesarean section and instrumental delivery, n), 2nd ring: EOCs by health care provider, 3rd ring: number of fatal (F, darkened) and non-fatal (NF) EOC birth outcomes by health care provider

With regard to the type of birth assistance, only deliveries without any birth assistance (2.4% of all deliveries) were more likely to terminate in stillbirth or early neonatal death (OR = 3.74; 95% CI 1.83–7.64) if compared to deliveries assisted by qualified medical personnel (48.8%). Assistance by family members/friends (34.6%), traditional birth assistants (11.9%), or other assistance (0.3%) than professional medical personnel did not show increased ORs for a fatal outcome in univariate regression analysis. Caesarean section and instrumental deliveries were associated with higher odds of fatal foetal outcomes (OR = 7.65; 95% CI 3.48–16.81 and OR = 5.12; 95% CI 2.02–13.03, respectively). Figure 3 illustrates numbers of such EOC measures undertaken and their fatality ratio and percentage in the general hospital of Taabo, the village-based health centres within and outside the Taabo HDSS. The overall percentage of fatal foetal events for caesarean section (eight out of 37; 21.6%) and instrumental delivery (five out of 33; 15.2%) were very high. The general hospital of Taabo showed a higher fatality ratio and percentage of fatal events from caesarean section (fatal (F):non-fatal (NF) ratio = 3:7; 30%), while health centres outside the Taabo HDSS showed a higher fatality ratio in instrumental delivery (F:NF ratio = 5:17; 23%) compared to zero fatal events (0:11; 0%) within the Taabo HDSS facilities.

One-fifth of all women reported to have experienced heavy bleeding during delivery, which was found to be associated with a negative impact on the foetal health (OR = 1.90; 95% CI 1.28–2.83) (Table 2). The mean cost of delivery was approximately FCFA 15,000 (approximately US$ 25). Our findings highlight a lower risk for fatal outcome in deliveries with assistance offered at a fee (irrespective of whether the amount is known (OR = 0.45; 95% CI 0.26–0.80) or not (OR = 0.44; 95% CI 0.28–0.68)), compared to those free of charge. Other potential factors, such as rubella, syphilis, and HIV testing, showed no significant association with foetal death in the univariate analysis.

### Pregnancy-related morbidities or concomitant health conditions and foetal outcome

Univariate logistic regression analysis showed several associations between pregnancy-related morbidities and other health conditions of expectant mothers and the foetal health outcome (Table 3). 7.8% of pregnant women included in the study had already experienced miscarriage or stillbirth in a previous pregnancy. For these women the risk for another fatal foetal outcome was strikingly high (OR = 22.34; 95% CI 15.01–33.25). Furthermore, morbidities of the genital tract, such as bleeding and discharge, had an influence on the foetal outcome. Women who had bleeding during pregnancy, be it in small quantities (2.7%; OR = 4.34; 95% CI 2.23–8.45) or accompanied by abdominal pain (3.5%; OR = 2.27; 95% CI 1.07–4.79), showed a higher odds to lose their child by miscarriage, stillbirth, or early neonatal death than their counterparts without bleeding. Foul-smelling vaginal discharge during pregnancy, which might indicate a genital infection, was reported by 20.9% of the women and was associated with increased odds for a fatal outcome (OR = 1.63; 95% CI 1.09–2.45).

**Table 3:** Pregnancy-related morbidities or concomitant health conditions in the Taabo HDSS in 2011–2014, stratified by foetal outcome, and crude ORs for fatal outcome from univariate logistic regression analysis

	Foetal outcome				
Characteristics	Live birth*	Fatal birth	Total		
	2858 (96.0)	118 (4.0)	2976 (100.0)	OR (95% CI)	*P*-value
Pregnancy-related morbidities or concomitant health conditions					
Earlier miscarriage/stillbirth					
No	2694 (94.3)	50 (42.4)	2744 (92.2)	1.00	
Yes	164 (5.7)	68 (57.6)	232 (7.8)	22.34 (15.01, 33.25)	< 0.001*
Vaginal bleeding during pregnancy					
No/cannot remember	2693 (94.2)	99 (83.9)	2792 (93.8)	1.00	
Yes, in small quantities	69 (2.4)	11 (9.3)	80 (2.7)	4.34 (2.23, 8.45)	< 0.001*
Yes, with preceding abdominal pain	96 (3.4)	8 (6.8)	104 (3.5)	2.27 (1.07, 4.79)	0.032*
Oedema of extremities or face					
No/cannot remember	2222 (77.7)	93 (78.8)	2315 (77.8)	1.00	
Yes	636 (22.3)	25 (21.2)	661 (22.2)	1.07 (0.68, 1.67)	0.785
Hypertension					
No/cannot remember	2829 (99.0)	116 (98.4)	2945 (99.0)	1.00	
Before pregnancy	13 (0.4)	1 (0.8)	14 (0.5)	0.49 (0.13, 1.79)	0.279
During pregnancy	16 (0.6)	1 (0.8)	17 (0.6)	0.62 (0.08, 5.01)	0.655
Persistent fever					
No/cannot remember	1769 (61.9)	63 (53.4)	1832 (61.6)	1.00	
Yes	1089 (38.1)	55 (46.6)	1144 (38.4)	1.42 (0.98, 2.05)	0.064
Malaria (reported or confirmed)					
No/cannot remember	1282 (44.9)	37 (31.4)	1319 (44.3)	1.00	
Yes	1576 (55.1)	81 (68.6)	1657 (55.7)	1.78 (1.20, 2.65)	0.004*
Urinary tract infection (dysuria)					
No/cannot remember	2508 (87.7)	92 (78.0)	2600 (87.4)	1.00	
Yes	350 (12.3)	26 (22.0)	376 (12.6)	2.03 (1.29, 3.17)	0.002*
Jaundice					
No/cannot remember	2461 (86.1)	91 (77.1)	2552 (85.7)	1.00	
Yes	397 (13.9)	27 (22.9)	424 (14.3)	1.84 (1.18, 2.86)	0.007*
Foul-smelling vaginal discharge					
No/cannot remember	2272 (79.5)	83 (70.3)	2355 (79.1)	1.00	
Yes	586 (20.5)	35 (29.7)	621 (20.9)	1.63 (1.09, 2.45)	0.017*
Gestational age					
≥37 weeks	2461 (86.1)	64 (54.2)	2525 (84.8)	1.00	
< 37 weeks	397 (13.9)	54 (45.8)	451 (15.2)	5.23 (3.59, 7.63)	< 0.001*

*CI* confidence interval, *OR* odds ratio

*Includes all live births with survival of more than 7 days

Concomitant diseases during pregnancy manifested a detrimental effect on the unborn child. Malaria (55.7%; OR = 1.78; 95% CI 1.20–2.65), jaundice (14.3%; OR = 1.84; 95% CI 1.18–2.86), and urinary tract infections (12.6%; OR = 2.03; 95% CI 1.29–3.17) ranked among the most important ones. Besides these morbidities and diseases, low gestational age (preterm birth at < 37 weeks, 15.2% of all births) was found a major risk factor for non-survival of the foetus (OR = 5.23; 95% CI 3.59–7.63). Known other co-morbidities during pregnancy, such as oedema, hypertension, and fever showed no significant relationship with foetal health in the Taabo HDSS during the 4-year observation period.

### Significant risk factors for fatal foetal outcome

Table 4 summarises all significant risk factors for a fatal foetal outcome, as revealed by multivariable logistic regression modelling. In terms of sociodemographic characteristics, women living in a rural area and from the poorest wealth tertile were at higher odds of experiencing foetal or neonatal death (aOR = 2.87; 95% CI 1.31–6.29 and aOR = 1.79; 95% CI 1.02–3.14, respectively). A prior history of miscarriage or stillbirth (aOR = 23.19; 95% CI 14.71–36.55) and giving birth by caesarean section (aOR = 13.03; 95% CI 4.24–40.08) or by instrumental delivery (aOR = 5.05; 95% CI 1.50–16.96) were major risk factors for an unfavourable foetal outcome in the adjusted model. Preterm birth (< 37 weeks of gestational age) showed a major impact on the health of the foetus after adjustment (aOR = 4.45; 95% CI 2.82–7.01). Further, payment for delivery was associated with a lower odds of fatal outcome (aOR = 0.39; 95% CI 0.25–0.74). Women not having received tetanus vaccination (aOR = 2.59; 95% CI 1.56–4.30) and women without iron/folic acid supplementation during pregnancy (aOR = 3.15; 95% CI 1.71–5.80) were at higher odds of miscarriage, stillbirth, or early neonatal death, compared to women covered by prevention efforts. With regard to pregnancy-related morbidities and concomitant health conditions, malaria during pregnancy was significantly associated with a fatal foetal outcome in the multivariable logistic regression model (aOR = 1.94; 95% CI 1.21–3.11). Vaginal bleeding of small quantities during pregnancy was no longer significantly associated with a fatal foetal outcome (*p* > 0.05) after adjustment to all other included covariates, while it remained a risk factor in the multivariable model (aOR = 1.98; 95% CI 0.94–4.15).

**Table 4:** Significant determinants for fatal foetal outcomes from multivariable regression analysis (adjusted for age, socioeconomic status and residency of the mother)

Explanatory variable^a^	aOR^b^	95% CI
Residency (rural)	2.87	1.31, 6.29
Socioeconomic status (poorest)	1.79	1.02, 3.14
Earlier miscarriage/stillbirth	23.19	14.71, 36.55
Delivery (by caesarean section)	13.03	4.24, 40.08
Delivery (instrumental)	5.05	1.50, 16.96
Gestational age (< 37 weeks)	4.45	2.82, 7.01
Delivery cost (chargeable)	0.39	0.25, 0.74
Absence of two doses of tetanus vaccination	2.59	1.56, 4.30
Absence of iron/folic acid supplementation	3.15	1.71, 5.80
Malaria (reported or confirmed)	1.94	1.21, 3.11

Multivariable logistic regression models using the penalized maximum likelihood estimation (Firth method) [27], to account for rare events, and a stepwise backward elimination approach were utilised to identify explanatory variables, which most significantly influence the foetal outcome. Initial models included (i) sociodemographic (i.e. age, socioeconomic status, and residency of the mother); (ii) birth circumstances and antenatal care; and (iii) pregnancy-related morbidity and concomitant health condition variables

Remaining explanatories were included at a significance level of *p* < 0.2

^a^Reference categories for explanatory variables: socioeconomic status, least poor; residency, urban; earlier miscarriage, none; delivery, normal; gestational age, ≥37 weeks; delivery cost, free of charge; two doses of tetanus vaccination, received; iron/folic acid supplementation, received; malaria (reported or confirmed), none

^b^Adjusted odds ratios

## Discussion

We present data pertaining to 4 years of carefully monitoring perinatal mortality and miscarriage from a primarily rural area in south-central Côte d’Ivoire. Our data stem from an HDSS, which is a well characterised population-based cohort that is subject to longitudinal surveillance and thus less prone to underreporting due to a lack of health system use or incomplete hospital-based registries. We found lower perinatal mortality and stillbirth rates compared to previous estimates for whole Côte d’Ivoire in 2004 and for the urban population of Abidjan some 20 years ago [30–32]. There are no recent studies on foetal and early neonatal death rates for Côte d’Ivoire, and hence, comparison with contemporary national-level data is not possible [33]. Our estimates of stillbirth rates are in line with rural settings elsewhere in sub-Saharan Africa that have been monitored in 2013 [34]. Furthermore, perinatal mortality, stillbirth, and early neonatal death rates for the Taabo HDSS are comparable to data obtained from 2002 to 2008 in neighbouring Ghana [35].

We identified a number of risk factors for an unfavourable foetal or early neonatal outcome, involving the expectant mothers’ sociodemographic characteristics, use of preventive measures and health services, and the experience of pregnancy-related or concomitant health conditions. Most of these factors are well known to impact negatively on foetal and neonatal health, such as a preterm birth [5, 36, 37] and a history of earlier miscarriage or stillbirth [38–40].

On first sight, the overall rate of pregnant women who went for antenatal care visits is high (96.3%). However, our data lack information on the exact number of visits, the gestation weeks at the time of the health visit, and the perceived and real quality of care received. A closer look at our data reveals that only a small proportion of pregnant women received and benefitted from standard prevention packages during such visits, such as a rubella test (4.8%), HIV testing (18.6%), iron/folic acid supplementation (28.0%), and two doses of tetanus vaccination (44.3%). These observations suggest either a generally low quality of provided antenatal care or the presence of stock-outs for material needed for essential interventions. If regular supplies are missing, they are sometimes still available at higher cost, reducing the number of women of reproductive age able to receive them. Another explanation is related to health seeking behaviour of the expectant mothers who visited antenatal care only once and probably at a late stage. Previous research suggests that antenatal care visits and prevention packages prevent against fatal foetal outcomes [30, 41, 42]. The low rate of prevention care received urges for a better coverage in the area and a deeper understanding of health care service use, since non-beneficiaries of the latter two measures were at higher risk for a fatal event.

In the Taabo HDSS and other rural areas of Côte d’Ivoire, infectious diseases are still the dominant cause of death [21]. Malaria due to *Plasmodium falciparum* is highly endemic [43–45] and our results demonstrate negative consequences on foetal health in mothers suffering from malaria. In Côte d’Ivoire, intermittent preventive treatment in pregnancy (IPTp) has been adopted since 2005. However, the effectiveness of protecting the mothers and unborn child from adverse events from malaria depend on the rigorous adherence of this policy, coverage, and the number of antenatal care visits of the expectant mothers [46, 47]. Furthermore, long-lasting insecticidal nets (LLINs) are available for malaria prevention. Bacterial infections, such as pneumonia and sepsis, are key drivers of neonatal death, as previously reported for the Taabo HDSS [21]. These deaths are preventable through administration of antibiotics during perinatal care and through strict observation of hygiene standards [46, 48]. Furthermore, early recognition and treatment is a key strategy for survival. Appropriate screening for urinary tract infections and preeclampsia, which are both known to have detrimental effects on foetal health [49, 50], is currently not feasible in the health district of Taabo. Both conditions could, however, be indirectly assessed as nitrite/leukocyte esterase and protein−/albuminuria using urine reagent strip analysis and, in case of preeclampsia, by specific blood pressure measurement to allow for early treatment [49, 51].

Our investigation also revealed a number of unexpected or contradicting findings that are offered for discussion. First, caesarean section is a surgical intervention to prevent or mitigate complications during pregnancy and childbirth. If applied timely and for appropriate indications, caesarean section is associated with lower incidence of stillbirth and early neonatal death [34, 52]. Among women with a fatal foetal outcome, 6.8% underwent a caesarean section, compared to only 1.0% among women with live birth. This finding, along with an overall low caesarean section rate of 1.2% (a proportion of 5–15% of births by caesarean section are considered as an indicator of acceptable EOC [48, 53]), may indicate that action is taken too late by the population and health services and reflect lack of access to, and malfunctioning of, health services. Of note, according to our data, more than half of all caesarean sections were undertaken in village health centres, however all these facilities do not dispose any operational block and these cases should thus have been referred to the Taabo hospital that is better equipped. While this finding might indicate some reporting mistakes during the questionnaire interviews about the final place of delivery, we cannot exclude any unofficially undertaken EOC by local health staff. A timely detection of malpresentations requiring caesarean section should be offered by midwifes. Conditions like placenta praevia and to a lesser degree foetal growth restriction as indications for early delivery would be crucial, but those imply the use of ultrasound [54], which was not available when our study was conducted.

In contrast to previous studies and international health efforts to effectively impact on new-born and maternal health [48, 55], non-facility-based delivery or non-professional birth assistance, with exception of no assistance at all, did not present a significant risk for pregnancy loss or neonatal death in our study cohort. This is important in light of half of all deliveries in the observation period being non-facility-based. This may partly be explained by more difficult cases rather being handled by professionals in facilities. However, even if more complicated cases that needed further instrumental intervention were excluded from the analysis, fatality rates for home deliveries were not significantly increased compared to deliveries taking place in health centres. While this may be a sign for traditional birth assistants working well, potentially referring critical cases to health facilities, it may be at the same time an indicator for insufficient quality of care in health centres. Our findings on higher numbers of fatal events among non-resident women using health facilities for delivery might indicate a selection bias with high-risk pregnancies (including referrals/transfers) or women with complications tending to use health facilities as would be desired, but health facilities unable to provide adequate care either due to late presentation or a poor level of care available. To promote facility-based deliveries may thus help to prevent late detection and arrival in the health centres of women with delivery complications, which is confirmed by our results of a lower odds for fatal outcomes in the urban population, characterised by a higher rate of facility-based deliveries compared to their rural counterparts (91.9% vs. 43.6%).

Both, the promotion of facility-based deliveries and coverage with preventive measures against congenital malformations and foetal malnutrition supplied during antenatal visits and against endemic infectious diseases impacting on maternal and foetal health require a deeper understanding of local drivers of health care use and traditional concepts of disease and pregnancy management among rural populations [16, 56]. A high risk among the poorest and rural dwellers may further indicate inequalities in access to, and use of, ante- and perinatal health care [57] that needs further scientific inquiry. In rural and often remote areas, community-based intervention packages, including training of traditional birth attendance, increase of coverage of preventive treatment, testing and nutritional supplementation as well as awareness campaigns for safe motherhood and neonatal health have been shown to positively impact on neonatal outcomes [42]. It is clear that the quality of receiving health services needs improvement in order to make them attractive, reduce access barriers, and enable them to provide appropriate, life-saving preventive and curative care.

Although we have tried to include a maximum number of potential factors influencing birth outcome, certain conditions known to be negatively related may have been missed since the current local health care infrastructure does not allow for its assessment or diagnosis (e.g. preeclampsia). A number of clinical factors, as assessed by our questionnaire administered to the expectant mothers, are based on self-reported symptomatology rather than determined through a clinical examination or a diagnostic device by a professional (e.g. malaria, jaundice, or urinary tract infection) and may thus lack accuracy. Likewise the estimation of gestational age in low-resource settings (including the current study) is prone to uncertainty since it depends on the capability of mothers to correctly recall their last menstrual period. Additionally, other information such as birth weight are often missing, and hence, estimates of gestational age are to be interpreted with care. In any event, mainly qualitatively-assessed clinical factors should be considered as proxies for the actual infection status, whereas for the gestational age we would like to highlight that we only used two categories; namely (i) pre-term and (ii) term. For relationship analysis, we considered the effect from potential misclassification of births close to this 37-week threshold as negligible for what was intended to be shown. With regard to the outcome measures, it is important to highlight that some of the so-called stillbirths may in fact be misclassified early neonatal deaths either from non-ability to distinguish between perinatal asphyxia or driven by cultural practices and beliefs favouring the interpretation of stillbirth as often observed in sub-Saharan Africa and thus introducing a social desirability bias into our analysis [58, 59]. Furthermore some pregnancies that ended in very early pregnancy loss may have been missed, and hence, the rate of miscarriage been underreported. Miscarriage is widely stigmatised, and hence, often remains a hidden phenomenon [60]. Nonetheless our study benefits from the use of population-based data that stem from an HDSS continuously registering vital events of more than 40,000 inhabitants. The monitoring of pregnancies is characterised by different stages whereas certain aspects are recorded in regular intervals during the course of the pregnancy, while others only get assessed once the outcome of the pregnancy is known. Our main outcomes such as miscarriage and perinatal mortality are thus less prone to underreporting compared to studies relying on hospital-based data repositories [36].

## Conclusion

Our data have shown that risk factors for fatal foetal outcomes in the Taabo HDSS are multifactorial, including the mother’s socioeconomic status and behaviour vis-à-vis prevention and use of antenatal care. Our results further confirmed predisposing conditions in expecting mothers such as earlier miscarriage or malaria as important factors to be considered. Hence, there is a pressing need for rigorous monitoring of high-risk pregnancies and early treatment of *Plasmodium* infection. Additionally, at the health system level, several aspects have been identified to influence birth outcomes, indicating the need to improve access and quality of care, especially in terms of early detection of complicated deliveries that could benefit from EOC. The evidence we provide may indicate how to most effectively tackle maternal and neonatal health through health system strengthening, community intervention packages and awareness campaigns on already existing services and policies for expectant mothers in the area. In a next step, however, local drivers for ante- and perinatal care use, compliance to preventive measures and community-identified needs for pregnant women in the current setting should be assessed, so that strategies can be tailored to reduce and prevent foetal and neonatal death.

## Abbreviations

aOR: Adjusted odds ratio
CHW: Community-health worker
CI: Confidence interval
CSRS: Centre Suisse de Recherches Scientifiques en Côte d’Ivoire
EOC: Emergency obstetric care
HDSS: Health and demographic surveillance system
INDEPTH: International Network for the continuous Demographic Evaluation of Populations and Their Health
IPTp: Intermittent preventive treatment in pregnancy
LLINs: Long-lasting insecticidal nets
LMP: Last menstrual period
OR: Odds ratio
PCA: Principal component analysis
Swiss TPH: Swiss Tropical and Public Health Institute
WHO: World Health Organization
